# Cardiac resynchronization therapy device implantation using suspended personal radiation protection system: Examination of radiation protection effectiveness by dosimetry at 51 exposure sites

**DOI:** 10.1016/j.hroo.2023.01.010

**Published:** 2023-02-02

**Authors:** Ryo Ito, Yusuke Kondo, Masahiro Nakano, Takatsugu Kajiyama, Yoshio Kobayashi

**Affiliations:** ∗ Department of Cardiovascular Medicine, Chiba University Graduate School of Medicine, Chiba, Japan; † Department of Advanced Cardiorhythm Therapeutics, Chiba University Graduate School of Medicine, Chiba, Japan

**Keywords:** Zero-Gravity, Suspended personal radiation protection system, CRT, Protection against radiation, Interventional cardiology

**Key Findings**

▪ A suspended personal radiation protection system, Zero-Gravity, is considered a novel approach that may overcome the concerns of conventional exposure protection devices, as it is a hanging type of protective equipment, which reduces the weight burden on the physician despite the sufficient lead content in the face shields and shoulders.
▪ In this study, we examined the radiation protection effectiveness of this system by dosimetry at 51 exposure sites during cardiac resynchronization therapy device implantation procedures.
▪ The absorbed doses inside this system, including the head, were below the lower limit of detection, suggesting a high radiation protection efficacy of the system.
▪ The Zero-Gravity system achieved high exposure protection for physicians even during cardiac resynchronization therapy device implantation.

## Introduction

Cardiologists often use fluoroscopic equipment for treatment such as cardiac implantable electronic device implantation, catheter ablation, percutaneous coronary intervention, and other procedures. Difficulties arise in protecting the head and optical lens from the exposure of radiation, which is a significant concern among physicians.^1^^,^^2^ Currently available radiation protective clothing consists of a collar for the thyroid gland and a sleeveless apron. Although higher radiation protection can be expected by increasing the lead content of the protective clothing, there is a limit to additional lead content due to the increased weight. Furthermore, goggles protecting against crystal exposure do not have a high lead content to ensure radiation protection. Shielding plates and other devices have been proposed to reduce the exposure dose; however, for some procedures such as device implantation, the disposition of the fluoroscopy equipment and the physician’s choice do not allow the application of a shielded plate. There are 2 main concerns. The first is maintaining a sterile environment of the fluoroscopy equipment to avoid infection, with the second being the physical positioning of the physician in order to perform the device implantation. In particular cardiac resynchronization therapy (CRT) device implantation increases the amount of time spent in the left anterior oblique position during fluoroscopy and contrast, which increases the physician’s exposure to radiation. Second, the weight of the radiation protective suit is not negligible and has also been implicated in the appearance of orthopedic symptoms such as back pain and shortened life expectancy of physicians.^3^

A suspended personal radiation protection system Zero-Gravity (TIDI Medical Products, Neenah, WI) is considered a novel approach that may overcome the concerns of conventional exposure protection devices, as it is a hanging type of protective equipment, which reduces the weight burden on the physician despite the sufficient lead content in the face shields and shoulders.^4^ We determined the extent to which the Zero-Gravity reduces radiation doses during CRT device implantation procedures.

## Methods

**Figure 1:**
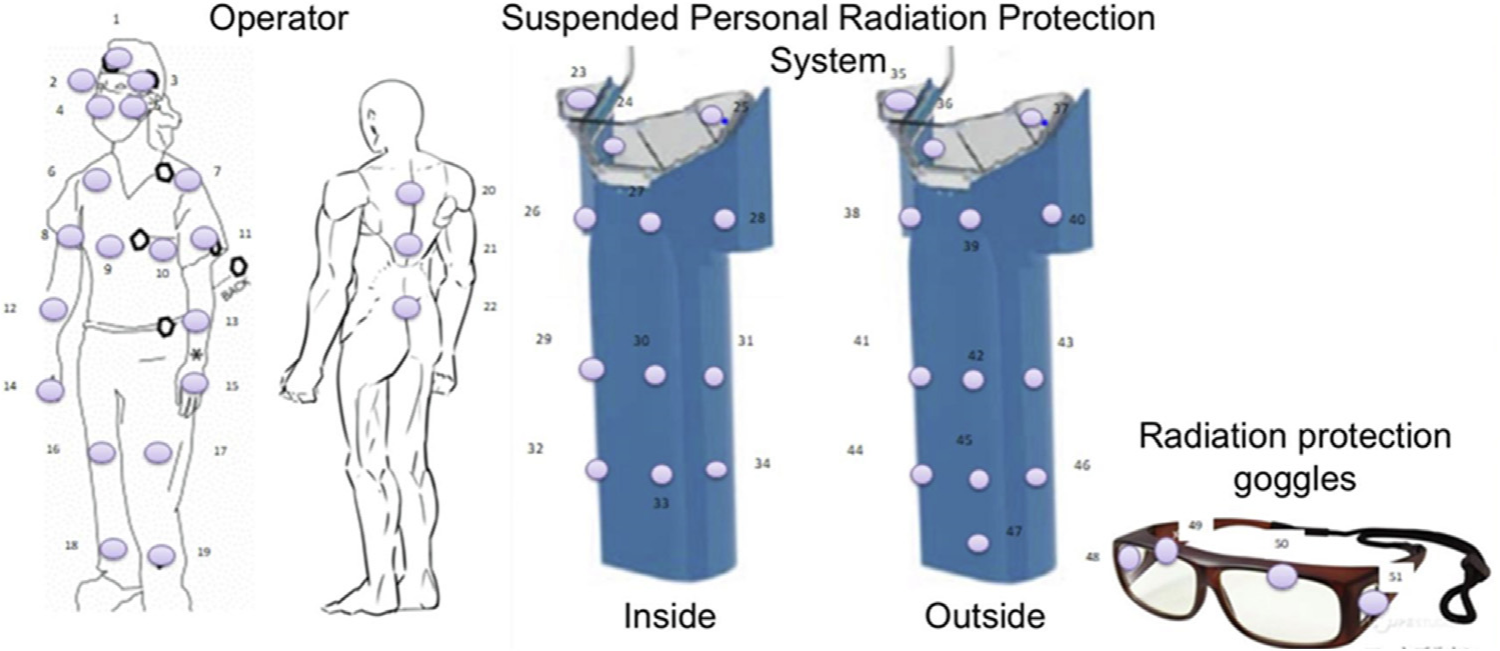
Attachment positions of dosimeters: on the frontal region, temporal regions, shoulders, upper arms, forearms, back of hands, things, and back of the operator; on the outside and inside of the suspended personal radiation protection system; and on the outside of the goggles.

**Figure 2:**
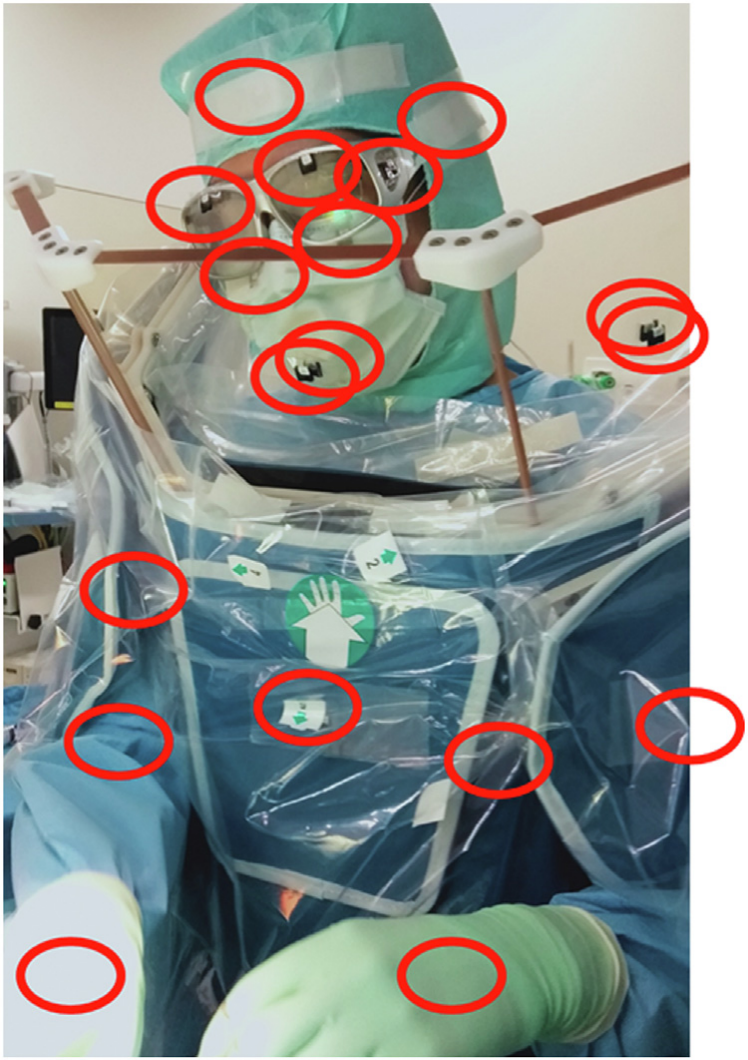
Photograph of the actual operator and the dosimeter attachment part.

Dosimeters were affixed to the physician's frontal, temporal, shoulder, upper arm, forearm, dorsal hand, thigh, lower leg, and back, as well as outside and inside the face guard of the Zero-Gravity. To measure exposure more accurately to the lens, the physician wore goggles and attached a dosimeter to the outside of the goggles. The nanoDot dosimeter (NAGASE-LANDAUER, Osaka, Japan) was used, while exposure dose measurement was performed using the microSTARii (NAGASE-LANDAUER). The SPRPS and the physician's dosimeters were attached to a total of 51 areas during CRT device implantation (Figures 1 and 2). The research reported in this article adhered to the ethical guidelines of the 1975 Declaration of Helsinki, and the study was approved by the Chiba University institutional review board; all patients provided written informed consent.

## Results

A CRT with defibrillator device was implanted in the left anterior thoracic region. The operation time was 2 hours and 6 minutes. The time required for left ventricular lead insertion was 43 minutes, with a total fluoroscopy time of 33 minutes and a total fluoroscopy volume of 58.05 mGy. Outside the Zero-Gravity area, the absorbed dose over the entire thigh was the largest at 0.4814 mGy, while the other doses tended to be higher on the right side because of its proximity to the fluoroscopy system. The absorbed doses inside the Zero-Gravity were below the detection limit. Physician exposure was higher in the unprotected hands, back, and elbows. Minimal exposure was observed in both the upper and forearms, but most was below the detection limit. The maximum absorbed dose outside of the goggles was 0.0064 mGy (Tables 1 and 2).

**Table 1:** Absorbed doses outside and inside of the suspended personal radiation protection system

Attached part of dosimeter	Absorbed dose outside the system (mGy)	Absorbed dose inside the system (mGy)
Neck shield (right side)	0.1301	0.0000
Neck shield (mid)	0.1166	0.0000
Neck shield (left side)	0.0364	0.0000
Right shoulder	0.0762	0.0000
Chest	0.1342	0.0000
Left shoulder	0.0000	0.0000
Right abdomen	0.1828	0.0000
Mid abdomen	0.2451	0.0000
Left abdomen	0.0000	0.0000
Right side of thigh	0.2641	0.0000
Mid side of thigh	0.4814	0.0000
Left side of thigh	0.0000	0.0000

**Table 2:** Absorbed dose to the operator and radiation protection goggles

Attached part of dosimeter	Absorbed dose to the operator (mGy)
Right upper arm	0.0015
Right forearm	0.1684
Right hand	0.2855
Left upper arm	0.0223
Left forearm	0.0620
Left hand	0.1440
All other parts	0.0000

Attached part of dosimeter	Absorbed dose outside the goggles (mGy)
Right eye	0.0064
Right temple	0.0000
Left eye	0.0010
Left temple	0.0043

## Discussion

During the CRT device implantation, the absorbed dose was the highest along the entire thigh surface, followed by the right hand and elbow. The fluoroscopy system irradiates from the bottom up; thus, the thighs are exposed to highest dose, and because the physician's right side is closer to the fluoroscopy system, this results in higher absorbed doses to the right hand, right elbow, and right side of the Zero-Gravity system. The absorbed doses inside this system, including the head, were below the lower limit of detection, suggesting a high radiation protection efficacy of the system. This is an important finding for reducing the risk of head and neck tumors due to head exposure to the interventionist. Regarding optical lens exposure, the absorbed dose ratio of the right side of the face shield and the right lens of the goggles showed a dose reduction of approximately 95%. Radiation-protective goggles are limited to a maximum dose reduction of 90%; thus, the Zero-Gravity was a more effective protection device. Previous reports have suggested that radiation protection is more effective using this system without a shielding plate than using conventional radiation protective clothing and a shielding plate in interventions.

## Conclusion

The Zero-Gravity system achieved high exposure protection for physicians even during CRT device implantation. Based on the results of this study, this system is expected to be highly effective as a radiation protection device not only during interventions, but also during CRT device implantation, in which it is more difficult to avoid exposure.
